# Ten-year helium anomaly prior to the 2014 Mt Ontake eruption

**DOI:** 10.1038/srep13069

**Published:** 2015-08-19

**Authors:** Yuji Sano, Takanori Kagoshima, Naoto Takahata, Yoshiro Nishio, Emilie Roulleau, Daniele L. Pinti, Tobias P. Fischer

**Affiliations:** 1 Atmosphere and Ocean Research Institute, The University of Tokyo, Kashiwa, Chiba, Japan; 2 Department of Geoscience, National Taiwan University, Taipei, Taiwan; 3 Research and Education Faculty, Kochi University, Nankoku, Kochi, Japan; 4 CEGA, Departamento de Geologia, Universidad de Chile, Santiago, Chile; 5 GEOTOP & Département des sciences de la Terre et de l’atmosphère, Université du Québec à Montréal, Montréal, Canada; 6 Department of Earth and Planetary Sciences, University of New Mexico, Albuquerque, New Mexico, USA

## Abstract

Mt Ontake in central Japan suddenly erupted on 27^th^ September 2014, killing 57 people with 6 still missing. It was a hydro-volcanic eruption and new magmatic material was not detected. There were no precursor signals such as seismicity and edifice inflation. It is difficult to predict hydro-volcanic eruptions because they are local phenomena that only affect a limited area surrounding the explosive vent. Here we report a long-term helium anomaly measured in hot springs close to the central cone. Helium-3 is the most sensitive tracer of magmatic volatiles. We have conducted spatial surveys around the volcano at once per few years since November 1981. The ^3^He/^4^He ratios of the closest site to the cone stayed constant until June 2000 and increased significantly from June 2003 to November 2014, while those of distant sites showed no valuable change. These observations suggest a recent re-activation of Mt Ontake and that helium-3 enhancement may have been a precursor of the 2014 eruption. We show that the eruption was ultimately caused by the increased input of magmatic volatiles over a ten-year period which resulted in the slow pressurization of the volcanic conduit leading to the hydro-volcanic event in September 2014.

Mt. Ontake is a strato-volcano (elevation 3067 m) located in central Honshu, Japan (35˚54’N, 137˚29’E). On 10^th^ September 2014, significant seismic activity was observed at the summit region. The number of seismic events decreased slightly in the following two weeks but remained at a considerably higher than background level^1^. The Japan Meteorological Agency did not release special instructions for the public because similar seismic activity was observed in 2011 without any eruption. Then the 27^th^ September 2014 Mt Ontake eruption occurred at 11:53 a.m., after an 11-minute period of tremor and uplift^2^. Several new craters were formed and large amounts of volcanic ash, rock and steam were ejected, producing a pyroclastic flow that traveled more than 3 km down the south flank and an ash plume that rose 7–10 km high. Ash-fall covered a large area, up to 50 cm thick near the craters. Juvenile magmatic material was not detected in the ash, suggesting that it was a hydro-volcanic eruption^3^. At the time of eruption, hundreds of hikers were in the region surrounding the crater and on the volcano’s slopes. Volcanic gas, ash and rocks killed fifty-seven people and 6 are still missing to date. It was the worst fatal eruption in postwar Japan history, exceeding the 43 killed in the 1991 eruption of Mt. Unzen in southern Japan^4^. It is difficult to predict hydro-volcanic eruptions because precursory phenomena are usually very scarce^5^ yet of the ~18,000 volcanic eruptions listed by the Global Volcanism Program about 5% (or 822) are assigned as hydro-volcanic^6^ and these types of eruptions have claimed approximately 20% of deaths related to historic eruptions^7^.

Helium-3 is the most important tracer among volatile species in volcanic-hydrothermal studies^8^,^9^ because of its mantle signature. Temporal variations of helium isotopes and volcanic activity are very well correlated, as shown in a steam well on the flanks of Izu-Oshima volcano, Japan^10^, in crater fumaroles at Galeras volcano, Colombia^11^, and in springs on the periphery of Mt. Etna, Italy^12^. Precursory changes of helium isotopes were reported also in fumarolic gases during the 2002–2003 eruption of Stromboli volcano, Italy^13^. All above helium isotopic anomalies were related to magmatic eruptions, none has yet been reported for hydro-volcanic eruptions. Here we show a ten-year helium anomaly related to the 2014 Mt Ontake eruption. A hydrodynamic dispersion model applied to the data provides an explanation for temporal variation of helium-3 flux at the conduit. The helium-3 flux can be converted into magmatic volatile flux, which may have led to the accumulation of steam in the volcanic edifice and the hydro-volcanic eruption.

## Results

### Helium isotopes and helium/neon ratios of gas samples

**Figure 1:**
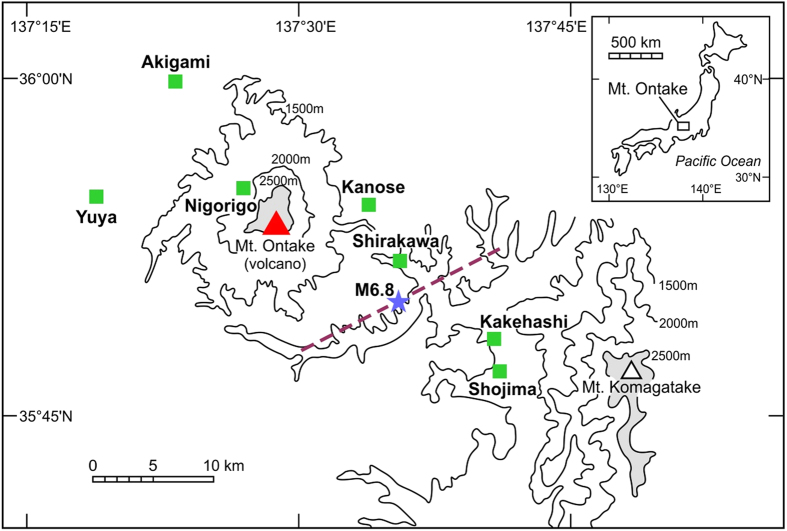
Location of sampling sites around Mt Ontake. Star and dotted line show the epicenter of the 1984 Western Nagano Earthquake and estimated fault line. Inset indicates regional location map. Original figure was retrieved from ref. 14 and modified by Yuji Sano.

We measured helium isotopes and helium/neon ratios of 92 gas samples in seven bubbling hot and minerals springs around Mt Ontake (Fig. 1). Samples were collected once every few years since November 1981^14^ (STable 1) and 12 samples were collected after the 2014 eruption. The ^3^He/^4^He and ^4^He/^20^Ne ratios vary significantly from 1.25 Ra to 7.38 Ra (where Ra is the atmospheric ratio^15^ of 1.382 × 10^−6^) and from 0.34 to 285 respectively. All helium isotopic ratios are higher than the air value, suggesting the influence of a mantle signature typical for arc volcanoes (7.4 ± 1.3 Ra^9^). Observed ^3^He/^4^He ratios are corrected for atmospheric contamination using helium/neon ratios^11^. Hereafter we use only corrected values, while we identified five samples collected between 1993 and 2007 with significant air contamination. During the whole observation period, the ^3^He/^4^He ratio generally decreases with increasing distance from the central cone to the sampling site (SFig. 1) suggesting that the most primitive magmatic ^3^He is carried with fluid flowing through the volcanic conduit^14^. As helium moves from the volcanic conduit through fissures and permeable channels to surrounding hot and mineral springs, the magmatic helium is diluted by radiogenic helium (0.02 Ra^16^) produced in aquifer rocks. This process results in lower ^3^He/^4^He ratios at more distant sites. However, monitoring of distant mineral springs still provides data that are, to a large extent, the direct result of variations of ^3^He/^4^He ratios in the volcanic conduit^10^,^11^,^12^.

### Secular variations of helium isotopes

**Figure 2:**
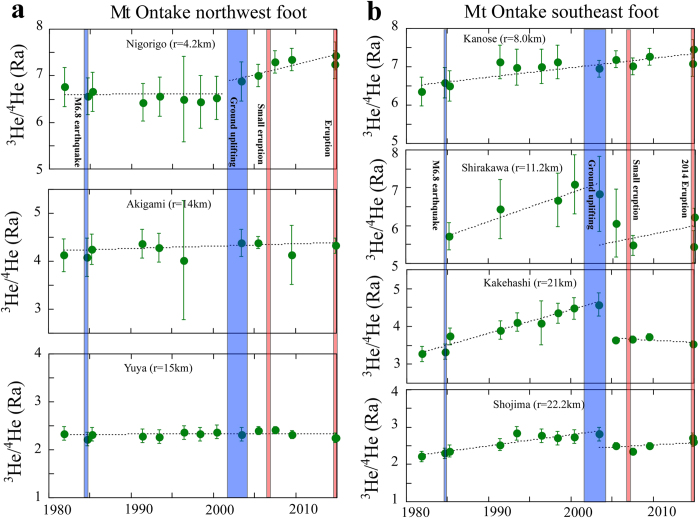
Temporal variations of corrected ^3^He/^4^He ratios at sampling sites around Mt Ontake. (**a**,**b**) show data of sites at northwest foot and southeast foot of the volcano, respectively. Blue and red bars indicate recent geotectonic events in the region.

Figure 2 shows secular variations of helium isotopes in seven natural springs where Fig. 2a indicates those in the northwest section of Mt Ontake and Fig. 2b those in the southeast. These data cover ^3^He/^4^He ratios of bubbling gas samples collected for 34 years since November 1981, comprising the longest record of hydrothermal helium isotope data in the noble gas literature^8^,^9^. In the northwest sites, ^3^He/^4^He ratios were generally constant within 2σ error from November 1981 to June 2000. Then the ratios increased significantly from June 2003 to November 2014 at Nigorigo hot spring, the closest site to the central cone. In contrast distant from the cone, the ratios stayed constant during the same period at Akigami and Yuya mineral springs (Fig. 2a). In the southeast sites, ^3^He/^4^He ratios were mostly variable (Fig. 2b). At the Kanose site close to the cone, the ratio increased gradually and with a constant rate from November 1981 to November 2014. On the other hand, there are two step changes of helium isotope values at Shirakawa, Kakehashi and Shojima, sites located relatively distant from the cone. The ^3^He/^4^He ratios increased significantly from November 1981 to June 2003 and then suddenly decreased and remained at a constant value until after the 2014 eruption. In summary spatial and secular variations of helium isotopes are complex and there is not a simple relationship except for recent increases of helium isotopes at Nigorogo site closest to the cone.

## Discussion

In order to study how the activation of Ontake volcano led to the fatal hydro-volcanic eruption, precise data analysis and hydro-geochemical modeling is necessary. In addition, the recent history of geotectonic events reported in the region is important for the interpretation of helium isotopes. These events are summarized as follows: The last magmatic activity was estimated to have occurred about 23,000 years ago^17^ and the volcano had been believed to be dormant, even though weak fumarolic activity was observed at the southwestern flank of the central cone. The first historical hydro-volcanic eruption occurred on 28^th^ October 1979, forming several new craters and ejecting large amounts of volcanic ash, rock and steam^18^. Five years later, a large earthquake (M6.8; the 1984 Western Nagano Earthquake) at shallow depth (2 km^19^) occurred about 10 km southeast of Mt Ontake on 14^th^ September 1984. Immediately after the earthquake, a large-scale landslide took place near the top of the volcano, killing 29 people on the southern slope. On 12^th^ November 1992, seismic activity occurred beneath the summit, followed by a white plume rising to 100 m above the crater^20^. Earthquake swarm activity had been observed since then in the region close to the M6.8 earthquake epicenter and ground uplift of 3–6 mm was detected from 2002 to 2004^21^. At the end of December 2006, intense seismic activity commenced beneath the summit of Mt Ontake^22^. A small hydro-volcanic eruption was identified by ash-fall deposits in the fumarole region in late March 2007^23^. All these observations indicate the reactivation of the volcano, but it remained quiescent from 2008 to 2014.

Figure 2b shows that there are significant temporal changes of helium isotopes in the southeast flank region of Mt Ontake (Shirakawa, Kakehashi and Shojima sites) during the period of the earthquake swarm and ground uplift from 2002 to 2004. General trends are similar among the three sites. ^3^He/^4^He ratios increased significantly after the 1984 Western Nagano Earthquake (M6.8) to before the uplift of 2002–2004. There is a negative relationship between the distance from the inferred earthquake fault (Fig. 1) and time rate of change (TROC) of helium isotopes at all seven sites between November 1981 and June 2003 (SFig. 2). Even though there may be a change of ^3^He/^4^He ratio within one year^24^, monotonic increases from 1981 and 2003 is a strong evidence of long-term mantle helium emanation at Shirakawa and Kakehashi sites. This correlation (SFig. 2) suggested that the M6.8 earthquake was induced by an upward migration of mantle fluids associated with diapiric magma intrusion beneath the source region^25^. Subsequent emission of mantle helium has ceased by the time of ground uplift, probably due to exhaustion of mantle volatiles in the small magma volume. Constant increase of ^3^He/^4^He ratio at Kanose site (Fig. 2b) may be due to a switch of the source of mantle helium from the diapiric magma in southeast flank to the central cone plumbing system during the time from 2002 to 2004. Different patterns at the sites Kanose and Shirakawa may be due to the distance from the fault (SFig. 2). Kanose is located further than Shirakawa and influence of diapiric magma may be smaller.

**Figure 3:**
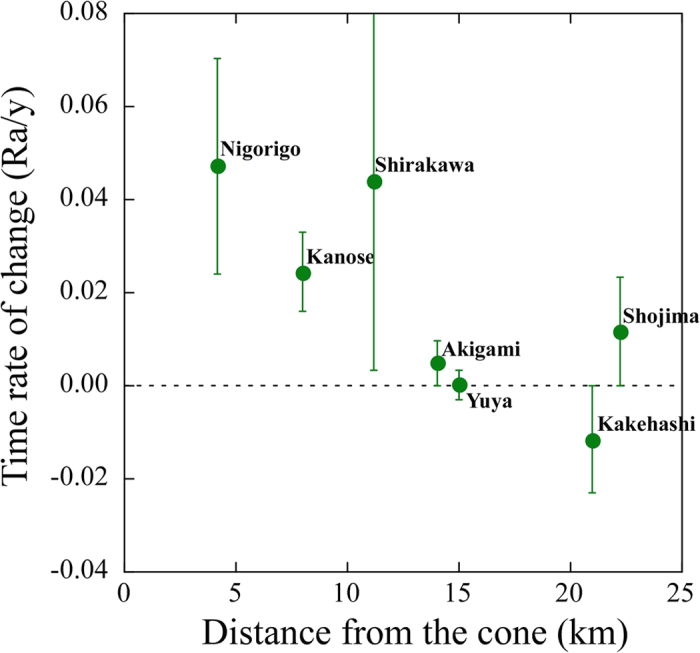
Correlation diagram between time rate of ^3^He/^4^He change since 2003 to 2014 and distance of the sampling site from the central cone of Mt Ontake.

Figure 3 indicates the relationship between the distance from the central cone of Mt Ontake and the TROC of helium isotopes after June 2003. There is a negative relationship between the distance and TROC, suggesting that the source of excess mantle helium is attributable to reactivated magma beneath the central crater. Decrease of crustal helium contribution into natural springs by aquifer rock dilatancy^26^ is not likely because there is not significant seismic activity in the northwest section. Therefore the recent ten years of increases in ^3^He/^4^He ratios at the Nigorigo and Kanose sites (Fig. 2) are mostly related to the central magma source of Mt Ontake, which may be related to the hydro-volcanic eruption.

**Figure 4:**
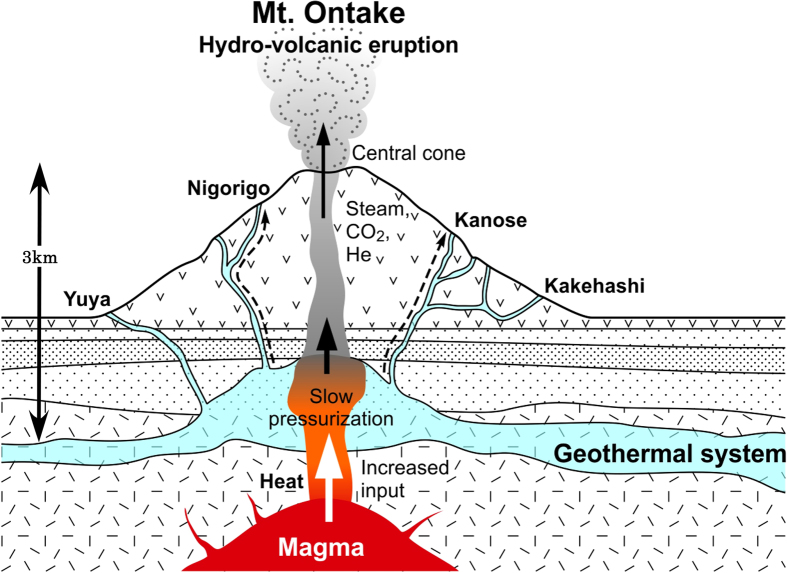
Schematic diagram of hydro-volcanic eruption of Mt. Ontake in September 2014. The increased input of magmatic volatiles resulted in slow pressurization of the volcanic conduit leading to the event. Depth scale was estimated from ref. 27. Original figure was created by Michelle Laithier of Université du Québec à Montréal.

There are two types of hydro-volcanic eruptions^5^; explosions of confined geothermal systems with or without the direct influence of magmatic fluids and those caused by the vaporization of surface fluids percolating into the temporarily plugged hot conduit of the volcano. The most likely cause of the 2014 eruption could be the former type of explosion because there is not a plugged hot conduit. Heating of shallow groundwater may have occurred during the magma rise, which may have increased the volatile pressure in the volcanic edifice. Ten years increase of helium isotopes at Nigorigo and Kanose sites suggests that the eruption process is slow (Fig. 4), caused by the gradual, rather than fast, accumulation of mantle volatiles during the rapid increase of volatile pressure produced by groundwater contact with the magma. Prior to the small hydro-volcanic eruption in March 2007, a very-long-period (VLP) volcanic event was detected by seismic observation^27^. The VLP event was explained as the response of a hydrothermal system to magma intrusion about 3 km beneath the summit of Mt Ontake. Therefore accumulation of volatile pressure was ongoing at least since 2007, which corresponds to the increase of helium isotope ratios at Nigorigo (Fig. 2).

To evaluate the risk of a possible hydro-volcanic eruption, it is important to study the rate of volatile input into the volcanic edifice. Monitoring of volcanic SO_2_ flux measurement may be useful to estimate this rate, but it has not been conducted at the central cone of Mt Ontake before the 2014 eruption. Using our data it is possible to estimate helium-3 flux at the conduit by a hydrodynamic dispersion model applied to the spatial variation of the helium isotopes in a given year^28^ (see Methods). Assuming that the fringe of the conduit is 1 km away from the center, which is the same size of the dike model^23^, secular variation of helium-3 flux can be estimated from 1981 to 2014 (SFig. 3). Calculated helium-3 flux is generally constant with a value of 2.86 ± 0.27 × 10^6^ atoms/m^2^sec (1σ error) from November 1981 to June 2003. Then the flux increased in June 2005 and stayed constant until June 2009 with the value of 3.56 ± 0.15 × 10^6^ atoms/m^2^sec. After the 2014 eruption, a flux of 2.82 ± 0.09 × 10^6^ atoms/m^2^sec was observed, i.e. the value before 2005. These calculations suggest that the magmatic activity may have decreased after the 2014 eruption. Even though the ^3^He/^4^He ratios of the Nigorigo site after the eruption were higher than those before June 2000, the overall helium variations explain the present calmness of the volcano.

Assuming that the depth of the aquifer is 30 m with an uncertainty of a factor of three and using the volcanic conduit diameter of 2 km, the hypothetical area of helium emission is 1.9 × 10^5^ m^2^ and the total helium-3 flux from the conduit of Mt Ontake before June 2003 is 78 nmol/day. The magmatic CO_2_/^3^He and H_2_O/CO_2_ ratios of high temperature subduction zone volcanic gases are well documented and summarized as 1 × 10^10^ and 100, respectively^29^. Using these values, the magmatic water flux is calculated as 1.4 tons/day based on the helium-3 flux and H_2_O/^3^He ratio. The magmatic water flux increased to 1.7 ton/day in June 2005 as the helium-3 flux was enhanced. This excess water supply of 0.3 ton/day, which likely continued over the last 10 years, led to an accumulated water amount of 1000 tons. This amount of water was introduced into the surrounding hydrothermal system and excess water vapor may have been trapped in the conduit just beneath the central cone (Fig. 4). This excess water vapor could have provided the driving force for the 2014 eruption.

In summary, we have observed a clear helium isotope increase at the hot spring close to Mt Ontake since June 2003, ten years before the 2014 fatal eruption. There were no consistent change at the distant sites. The helium anomaly is likely related to the recent activation of magma and is valuable for the mitigation of volcanic hazard in future.

## Methods

### Sampling, analysis and data reduction

Hot and mineral spring gases were collected by water displacement method using an inverted funnel, a manual pump and a lead glass container^9^. All sampling sites are natural springs and we did not use any lifting pump system. A portion of gas sample was introduced into a metallic high vacuum line in the laboratory, where helium and neon were purified by hot Ti getters and charcoal traps at liquid nitrogen temperature. Then the ^4^He/^20^Ne ratios were measured by a quadrupole mass spectrometer and helium was separated from neon by a cryogenic charcoal trap. Samples before 1990 and 2003 were measured by a Nuclide noble gas mass spectrometer without separating helium from neon^30^, while those after 1990 except for 2003 were analyzed by a VG5400 mass spectrometer^31^. There is an experimental bias of about 9% between the two systems. However the difference was well corrected by a careful treatement^32^,^33^. Samples collected after the 2014 eruption were measured by the same system as for the 1993–2009 samples. Therefore there is no bias expected among them. Correction of the ^3^He/^4^He ratio for air contamination was made based on the ^4^He/^20^Ne ratio. If the ^4^He/^20^Ne ratio is close to the air value, the correction could be significantly erroneous^11^. Therefore we masked five samples with low ^4^He/^20^Ne ratios (STable 1).

### Hydrodynamic dispersion model

In order to explain the observed helium isotope trend around the volcano (SFig. 1), a hydrodynamic dispersion model was developed^28^ Assuming that thermal fluids are supplied from a magma reservoir to the conduit at a constant rate, and that the boundary conditions are such that the height of the piezometric head has the same distribution in any vertical section through the axis of the conduit, it is possible to estimate the fluid flow and thus helium isotopes based on the dispersion model. The equation governing helium isotopes at distance (r) under steady-state, homogeneous and isotropic conditions is as follows:


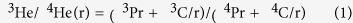


where ^3^P, ^4^P, ^3^C and ^4^C denote nucleogenic and radiogenic production rate of ^3^He and ^4^He, hypothetical concentration of ^3^He and ^4^He at conduit, respectively. Assuming typical sedimentary material composing the aquifer, ^3^P and ^4^P is 1.5 × 10^6^ atoms/m^3^sec and 3 × 10^−2^  atoms/m^3^sec, respectively. It is possible to calculate ^3^C and ^4^C values by fitting the observed helium isotope distribution to the above equation by the least-squares method. Despite the model being simplistic, it reproduced well the spatial distribution of helium isotopes at several volcanoes (Mt Nevado del Ruiz, Mt Hakone, Mt Kusatsu and Mt Unzen)^9^. The method is applied to the spatial data set of year 1981, 1984, 1985, 1991, 1993, 1996, 1998, 2003, 2005, 2007, 2009, and 2014. It is difficult to calculate the data of 2000 because the number of data is too small. Helium-3 flux at the conduit is estimated by the term of “^3^C/r” in above equation for each year. Secular variation of the helium-3 flux is plotted in SFig. 3 where the error is 2 sigma obtained by the least-squares method.

## Additional Information

**How to cite this article**: Sano, Y. *et al.* Ten-year helium anomaly prior to the 2014 Mt Ontake eruption. *Sci. Rep.* **5**, 13069; doi: 10.1038/srep13069 (2015).

## Supplementary Material

Supplementary Information
