# Supplementary material for: Ten-year helium anomaly prior to the 2014 Mt Ontake eruption
Source: Sci Rep. 2015 Aug 19;5:13069. doi: 10.1038/srep13069 (PMC4541341; doi:10.1038/srep13069)
Supplement: Supplementary Information [file srep13069-s1.pdf]

Supplementary Information

Ten-year helium anomaly prior to the 2014 Mt Ontake eruption

Yuji Sano[1,2]*, Takanori Kagoshima[1], Naoto Takahata[1], Yoshiro Nishio[3], Emilie Roulleau[4], Daniele L. Pinti[5] and Tobias P. Fischer[6]

1. Atmosphere and Ocean Research Institute, The University of Tokyo, Kashiwa, Chiba, Japan.
2. Department of Geoscience, Taiwan National University, Taipei, Taiwan.
3. Research and Education Faculty, Kochi University, Nankoku, Kochi, Japan.
4. CEGA, Facultad de Ciencias Físicas y Matemáticas, Universidad de Chile, Santiago, Chile.
5. GEOTOP & Département des sciences de la Terre et de l'atmosphère, Université du Québec à Montréal, Montréal, Canada.
6. Department of Earth and Planetary Sciences, University of New Mexico, Albuquerque, New Mexico, USA.

*Correspondence and requests for materials should be addressed to Y.S. (e-mail: ysano@aori.u-tokyo.ac.jp)

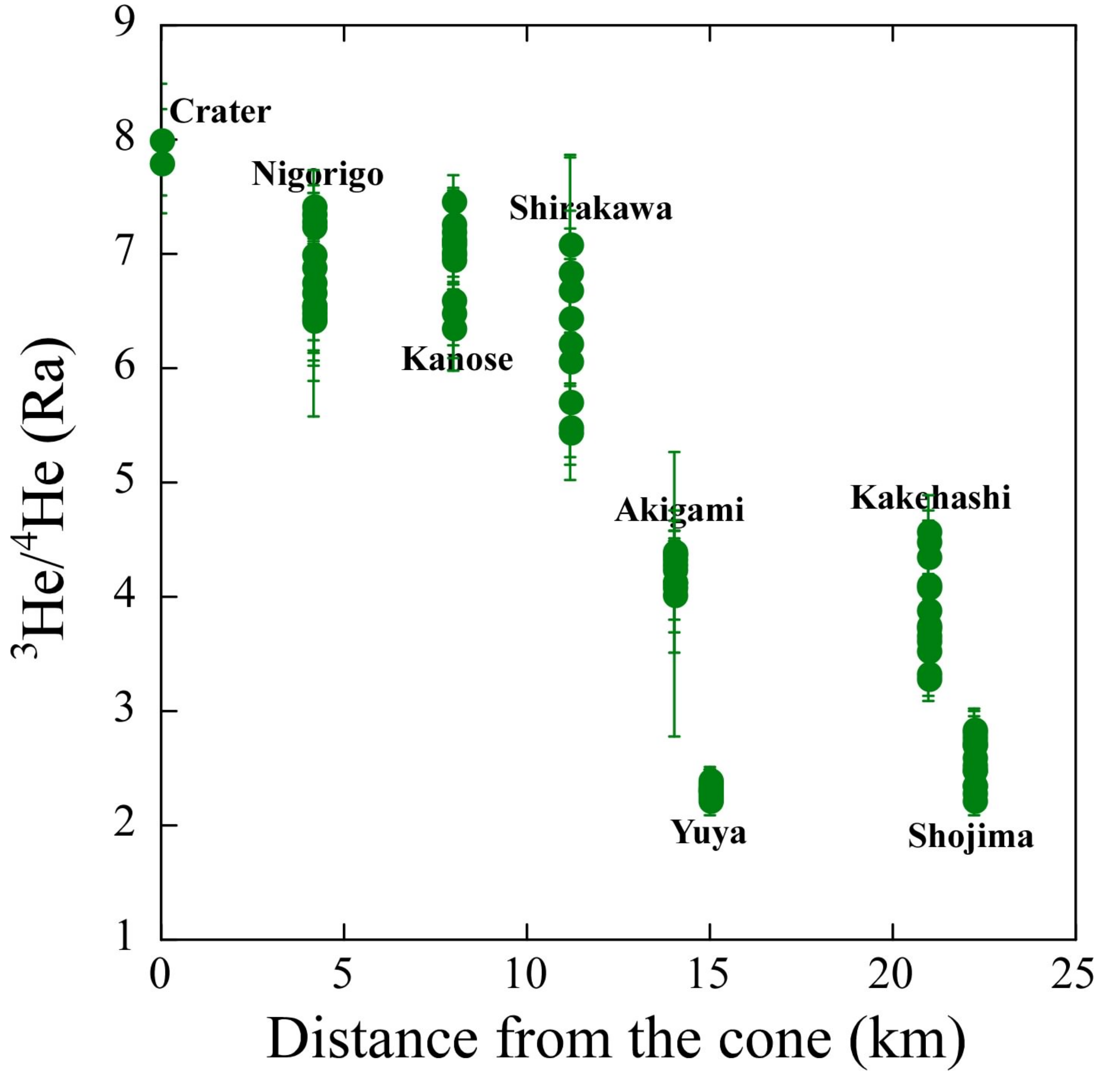

Supplementary Figure 1. Correlation diagram between the distance of the sampling site from the central cone of Mt Ontake and the corrected $^3He/^4He$ ratio. Data before 2000 are from ref. 32 and 33.

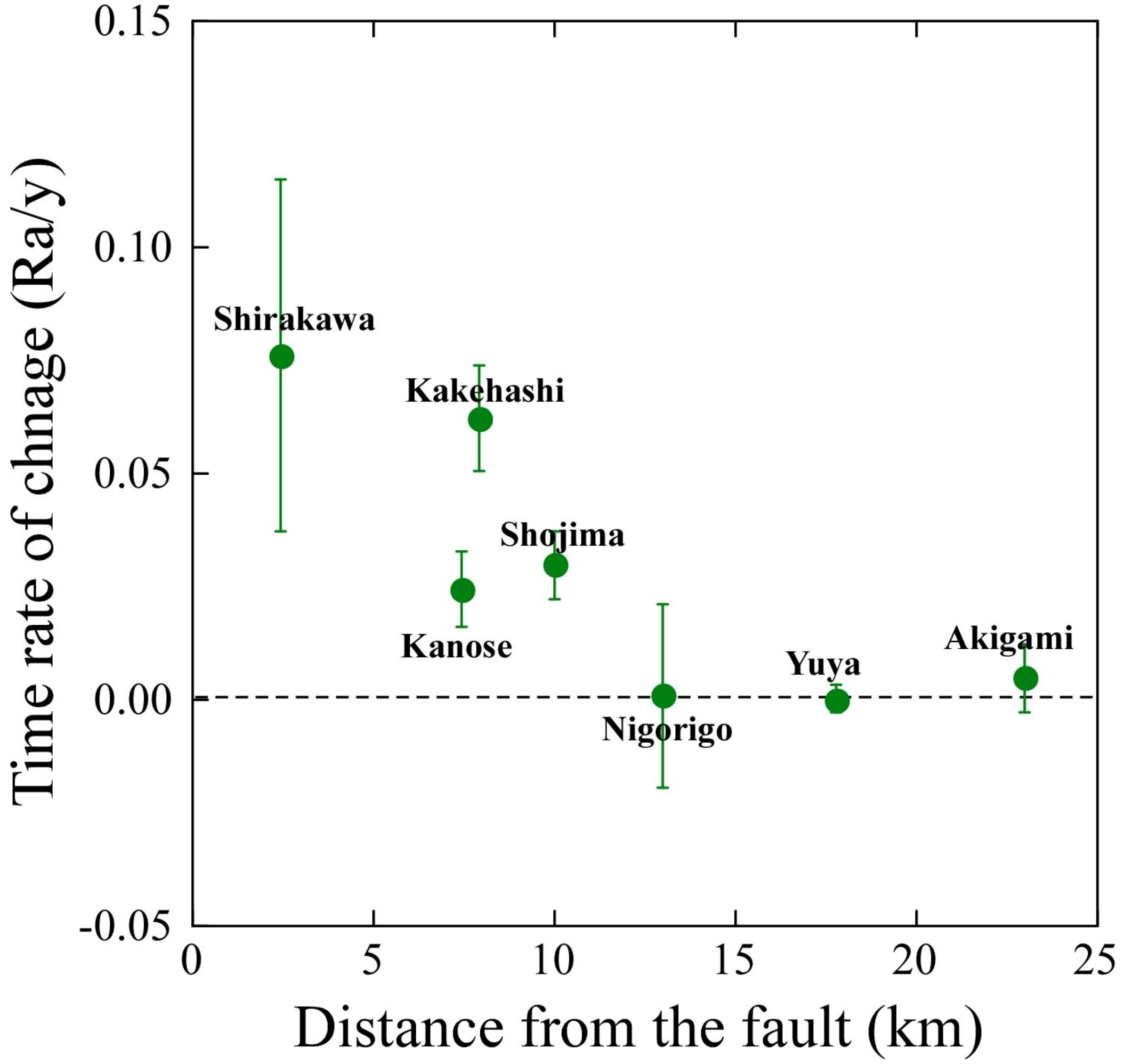

Supplementary Figure 2. Correlation diagram between time rate of $^3He/^4He$ change since 1981 to 2003 and distance of the sampling site from the estimated fault line of the 1984 Western Nagano Earthquake.

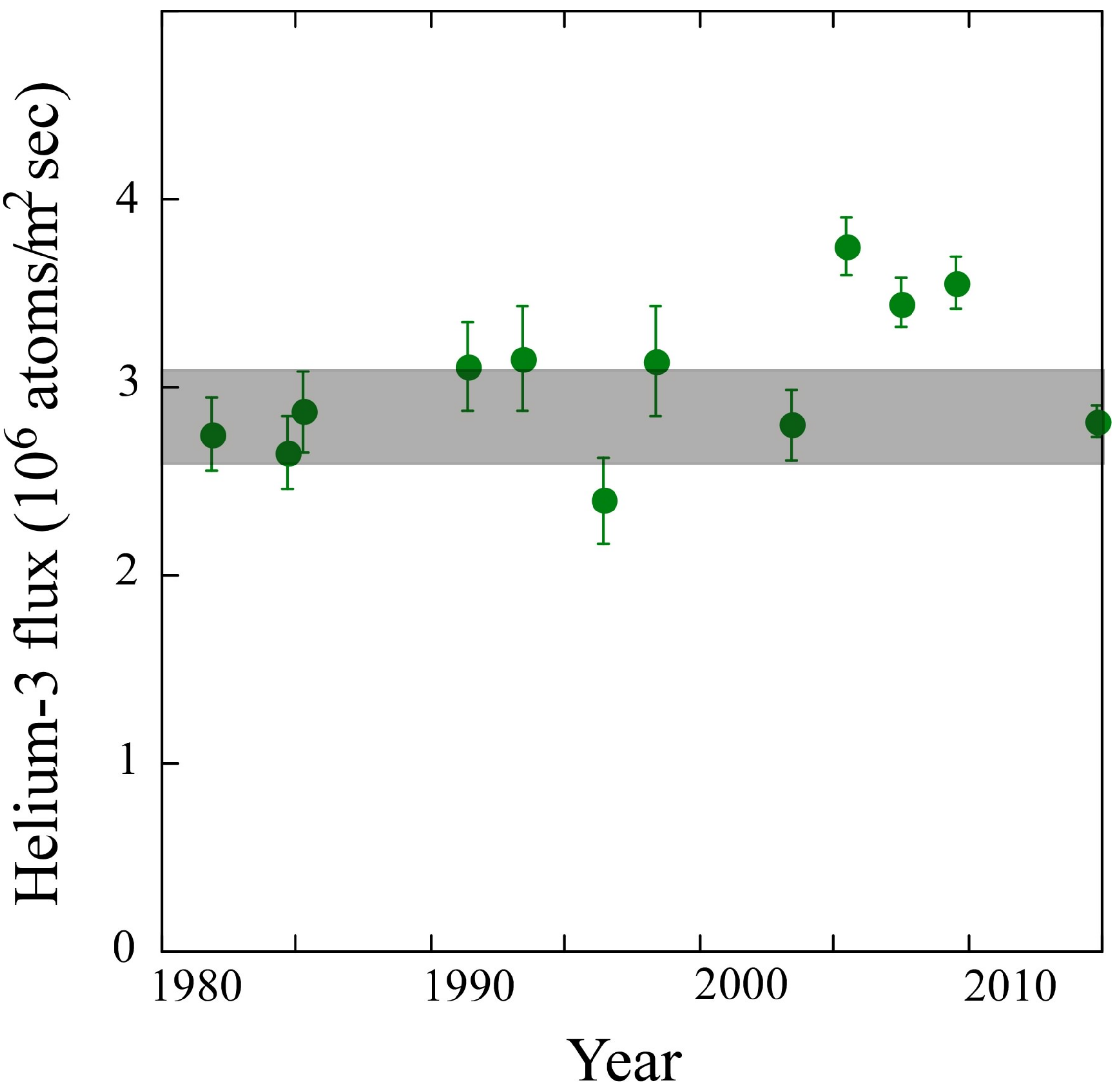

Supplementary Figure 3. Temporal variations of estimated helium-3 flux at 1 km away from the central cone. Shade shows the average of the flux from 1981 to 2003 with one sigma error. After the 2014 eruption, the flux returned to the value before 2005.

Supplementary Table 1. Observed $^3He/^4He$, $^4He/^{20}Ne$ and corrected $^3He/^4He$ ratios.

Name	Date	$^3He/^4He$ (Ra)	$^4He/^{20}Ne$	$^3He/^4He$ corr (Ra)	Error (2σ)
Akigami	3-Nov-81	3.44	2.5	4.14	0.34
Akigami	22-Sep-84	3.17	1.5	4.09	0.41
Akigami	1-Apr-85	3.62	3.3	4.25	0.32
Akigami	3-Jun-91	4.23	7.9	4.37	0.29
Akigami	9-Jun-93	3.76	5.5	4.28	0.30
Akigami	7-Jun-96	2.04	0.52	4.02	1.23
Akigami	5-Jun-00	1.55	0.39	4.28*	4.11*
Akigami	3-Jun-03	3.98	24	4.38	0.27
Akigami	21-Jun-05	4.19	5.5	4.39	0.13
Akigami	25-Jun-07	1.25	0.34	4.40*	4.85*
Akigami	29-Jul-09	2.01	0.47	4.13	0.62
Akigami	13-Nov-14	3.67	1.6	4.33	0.15
Kakehashi	3-Nov-81	2.98	23	3.28	0.20
Kakehashi	23-Sep-84	3.04	41	3.33	0.20
Kakehashi	2-Apr-85	3.43	96	3.75	0.23
Kakehashi	4-Jun-91	3.89	150	3.90	0.24
Kakehashi	10-Jun-93	3.76	82	4.11	0.25
Kakehashi	8-Jun-96	2.77	0.89	4.09	0.59
Kakehashi	1-Jun-98	3.99	107	4.35	0.26
Kakehashi	4-Jun-00	4.09	44	4.48	0.27
Kakehashi	3-Jun-03	4.06	7.1	4.58	0.31
Kakehashi	20-Jun-05	3.60	27	3.63	0.08
Kakehashi	26-Jun-07	3.64	37	3.67	0.08
Kakehashi	30-Jul-09	3.71	48	3.73	0.08
Kakehashi	14-Nov-14	3.46	10	3.54	0.09
Kanose	2-Nov-81	5.81	83	6.35	0.38
Kanose	22-Sep-84	6.03	92	6.59	0.40
Kanose	2-Apr-85	5.95	91	6.50	0.39
Kanose	3-Jun-91	7.08	51	7.12	0.43
Kanose	10-Jun-93	6.20	8.0	6.99	0.47
Kanose	7-Jun-96	6.38	31	7.01	0.43
Kanose	2-Jun-98	6.48	27	7.13	0.44
Kanose	4-Jun-00	2.60	0.46	6.78*	3.29*
Kanose	3-Jun-03	6.34	44	6.95	0.23
Kanose	20-Jun-05	7.15	53	7.19	0.22
Kanose	26-Jun-07	6.96	32	7.02	0.22
Kanose	30-Jul-09	7.20	29	7.27	0.22
Kanose	7-Oct-14	6.99	17	7.10	0.34
Kanose	14-Nov-14	7.33	16	7.46	0.24

Supplementary Table 1. (continued)

Name	Date	$^3He/^4He$ (Ra)	$^4He/^{20}Ne$	$^3He/^4He$ corr (Ra)	Error (2σ)
Nigorigo	2-Nov-81	6.15	32	6.76	0.42
Nigorigo	23-Sep-84	6.00	79	6.56	0.40
Nigorigo	1-Apr-85	6.06	29	6.66	0.41
Nigorigo	3-Jun-91	6.39	39	6.43	0.40
Nigorigo	9-Jun-93	5.92	15	6.56	0.42
Nigorigo	7-Jun-96	4.37	0.99	6.50	0.92
Nigorigo	1-Jun-98	5.21	2.2	6.44	0.57
Nigorigo	5-Jun-00	5.71	5.6	6.53	0.46
Nigorigo	4-Jun-03	6.27	39	6.88	0.42
Nigorigo	21-Jun-05	6.54	4.2	7.00	0.24
Nigorigo	26-Jun-07	7.23	32	7.29	0.23
Nigorigo	29-Jul-09	7.25	21	7.35	0.24
Nigorigo	8-Oct-14	7.17	28	7.24	0.30
Nigorigo	13-Nov-14	7.38	42	7.43	0.31
Shirakawa	1-Apr-85	5.18	22	5.71	0.36
Shirakawa	4-Jun-91	5.00	1.20	6.44	0.79
Shirakawa	10-Jun-93	2.06	0.40	6.75*	6.22*
Shirakawa	7-Jun-96	3.20	0.58	6.40*	1.74*
Shirakawa	2-Jun-98	5.09	1.57	6.68	0.69
Shirakawa	4-Jun-00	5.25	1.40	7.09	0.79
Shirakawa	3-Jun-03	4.53	0.96	6.84	0.99
Shirakawa	20-Jun-05	4.00	0.78	6.06	0.90
Shirakawa	26-Jun-07	5.05	3.32	5.48	0.26
Shirakawa	9-Oct-14	4.65	1.77	5.45	0.42
Shirakawa	14-Nov-14	6.08	11.6	6.22	0.23
Shojima	3-Nov-81	2.02	17	2.22	0.14
Shojima	22-Sep-84	2.07	9.3	2.30	0.15
Shojima	2-Apr-85	2.05	3.2	2.36	0.17
Shojima	3-Jun-91	2.52	61	2.53	0.15
Shojima	9-Jun-93	2.54	7.9	2.84	0.19
Shojima	8-Jun-96	2.51	15	2.77	0.17
Shojima	2-Jun-98	2.42	6.9	2.71	0.18
Shojima	5-Jun-00	2.47	11	2.74	0.17
Shojima	3-Jun-03	2.56	18	2.82	0.18
Shojima	20-Jun-05	2.44	8.1	2.50	0.09
Shojima	26-Jun-07	2.33	14	2.36	0.08
Shojima	30-Jul-09	2.46	13.6	2.50	0.08
Shojima	8-Oct-14	2.63	6.9	2.71	0.13
Shojima	12-Nov-14	2.56	10.8	2.61	0.11

Supplementary Table 1. (continued)

Name	Date	$^3He/^4He$ (Ra)	$^4He/^{20}Ne$	$^3He/^4He$ corr (Ra)	Error (2σ)
yuya	21-Nov-81	2.14	37	2.34	0.14
yuya	23-Sep-84	2.04	110	2.23	0.13
yuya	1-Apr-85	2.13	99	2.32	0.14
yuya	4-Jun-91	2.29	120	2.29	0.14
yuya	10-Jun-93	2.08	285	2.27	0.14
yuya	7-Jun-96	2.16	54	2.36	0.14
yuya	1-Jun-98	2.14	113	2.33	0.14
Yuya	4-Jun-00	2.17	53	2.37	0.14
Yuya	4-Jun-03	2.13	60	2.32	0.14
Yuya	21-Jun-05	2.39	40	2.40	0.07
Yuya	25-Jun-07	2.40	38	2.41	0.07
Yuya	29-Jul-09	2.30	21	2.32	0.07
Yuya	8-Oct-14	2.24	47	2.25	0.09
Yuya	13-Nov-14	2.24	43	2.25	0.09

Data before 2000 are listed in Takahata et al. (2003) Geochemical Journal 37, 299-310 and in Sano et al. (1998) Journal of Geophysical Research 103, 23863-23873.

*: masked because of air contamination.
